# Radical Prostatectomy: Sequelae in the Course of Time

**DOI:** 10.3389/fsurg.2021.684088

**Published:** 2021-05-28

**Authors:** Claudia Kesch, Isabel Heidegger, Veeru Kasivisvanathan, Alexander Kretschmer, Giancarlo Marra, Felix Preisser, Derya Tilki, Igor Tsaur, Massimo Valerio, Roderick C. N. van den Bergh, Christian D. Fankhauser, Fabio Zattoni, Giorgio Gandaglia

**Affiliations:** ^1^Department of Urology, University Hospital Essen, Essen, Germany; ^2^Department of Urology, Medical University Innsbruck, Innsbruck, Austria; ^3^Division of Surgery and Interventional Science, University College London, London, United Kingdom; ^4^Department of Urology, University College London Hospital, London, United Kingdom; ^5^Department of Urology, Ludwig-Maximilians-University of Munich, Munich, Germany; ^6^Department of Urology, San Giovanni Battista Hospital, University of Turin, Turin, Italy; ^7^Department of Urology, University Hospital Frankfurt, Frankfurt, Germany; ^8^Martini-Klinik Prostate Cancer Center, University Hospital Hamburg-Eppendorf, Hamburg, Germany; ^9^Department of Urology, University Hospital Hamburg-Eppendorf, Hamburg, Germany; ^10^Department of Urology and Pediatric Urology, Mainz University Medicine, Mainz, Germany; ^11^Department of Urology, Centre Hospitalier Universitaire Vaudois (CHUV) Lausanne, Lausanne, Switzerland; ^12^Department of Urology, Antonius Hospital, Utrecht, Netherlands; ^13^Department of Urology, University Hospital Zürich, Zurich, Switzerland; ^14^Urology Unit, Azienda Sanitaria Universitaria Integrata di Udine, Udine, Italy; ^15^Division of Oncology/Unit of Urology, Urological Research Institute, Istituto di Ricovero e Cura a Carattere Scientifico (IRCCS) Ospedale San Raffaele, Milan, Italy

**Keywords:** prostate cancer, retropubic radical prostatectomy, robot-assisted radical prostatectomy, adverse (side) effects, long-term outcome

## Abstract

**Objective:** Radical prostatectomy (RP) is a frequent treatment for men suffering from localized prostate cancer (PCa). Whilst offering a high chance for cure, it does not come without a significant impact on health-related quality of life. Herein we review the common adverse effects RP may have over the course of time.

**Methods:** A collaborative narrative review was performed with the identification of the principal studies on the topic. The search was executed by a relevant term search on PubMed from 2010 to February 2021.

**Results:** Rates of major complications in patients undergoing RP are generally low. The main adverse effects are erectile dysfunction varying from 11 to 87% and urinary incontinence varying from 0 to 87% with a peak in functional decline shortly after surgery, and dependent on definitions. Different less frequent side effects also need to be taken into account. The highest rate of recovery is seen within the first year after RP, but even long-term improvements are possible. Nevertheless, for some men these adverse effects are long lasting and different, less frequent side effects also need to be taken into account. Despite many technical advances over the last two decades no surgical approach can be clearly favored when looking at long-term outcome, as surgical volume and experience as well as individual patient characteristics are still the most influential variables.

**Conclusions:** The frequency of erectile function and urinary continence side effects after RP, and the trajectory of recovery, need to be taken into account when counseling patients about their treatment options for prostate cancer.

## Introduction

With an age-standardized incidence rate of 30.7 per 100.00, prostate cancer (PCa) is the second most frequent cancer excluding non-melanoma skin cancer in men worldwide (1). Radical prostatectomy (RP) is one of the main treatment options for these men and its frequency has increased and evolved rapidly since the 1980s (2). The first successful open RP was performed in 1904 by Hugh Hampton Young and William Stewart Halsted at the Johns Hopkins Hospital in Baltimore, USA using a perineal approach (3). It took another 40 years for the first series of retropubic prostatectomies being published by Terence Millin in 1945 (3). Thanks to Patrick Walsh's detailed description of the cavernous nerves and the dorsal venous complex enabling a nerve sparing technique and better surgical control in 1982, retropubic RP finally gained popularity becoming the preferred technique (4). Aiming to reduce postoperative morbidity and allowing faster recovery the first laparoscopic RP (LRP) was performed in 1997 (4). However, surgeons adopting LRP were facing technical and ergonomic challenges and needed to overcome a significant learning curve prior to achieving similar results to experienced open surgeons (5). Addressing the technical limitations of LPR, robot-assisted RP (RARP) was introduced in the early 2000s by Claude Abbou and Jochen Binder using the da Vinci Surgical System® (6, 7) and has by now become the preferred minimally-invasive approach. Whilst RP offers a cure for many men suffering from PCa where the recurrence rates are around 20% at 5-year follow-up (8), this surgical approach does not come without significant short- and long-term adverse effects, with decline in sexual function and urinary incontinence being the ones most frequently reported. However, due to the implementation of different, mostly minimally invasive techniques and a lack of standardized reporting of surgical complications for RP there is a wide variation in incidences and types of complications reported. This review aims to assess the current literature in regard to the sequelae of RP over the course of time, focusing on studies that include key domains recommended by international groups (9–11) and are following reporting guidelines (12).

## Evidence Acquisition

A collaborative literature research was performed by a relevant term search on PubMed from 2010 to 9th of February 2021, identifying recently published randomized and non-randomized studies where outcome data were collected, data acquisition was performed mostly prospective after primary RP for PCa and outcomes were measured by validated patient-reported outcome measures (PROMS) (12) with mostly at least 12 month of follow up. The medical electronic database Pubmed was searched using keywords: “radical prostatectomy” AND/OR “outcomes” AND/OR “health related quality of life” AND/OR “adverse effects” AND/OR “long-term outcomes” AND/OR “open radical prostatectomy vs. robot-assisted radical prostatectomy.” The identified studies represented the basis for a narrative review of the literature.

## Evidence

### Sexual Function

Post RP erectile function decline is a major postoperative complication and can have a great impact on the quality of life of the patient. Risk factors for postoperative erectile dysfunction (ED) include non-nerve sparing surgery, the surgeon's learning curve, age of the patient, baseline sexual function, diabetes, hypertension, and smoking (13). A recently published study on patient reported outcomes through 5 years after therapy for localized PCa evaluated sexual function, amongst others, using the validated 26-item Expanded Prostate Index Composite (EPIC) (14, 15). They found a clinically meaningful decline [validated minimum clinically important difference (MCID), 10–12] in sexual function for both, patients with favorable (cT1 to cT2b, PSA ≤ 20 ng/ml, ISUP 1–2) and unfavorable-risk disease (≥cT2c, PSA 20–50 ng/l, ISUP 1–5). On a score scale ranging from 0 to 100, with higher scores indicating fewer symptoms and dysfunction, in men with unfavorable-risk disease (*n* = 402), a decline from a baseline domain score of 70 to 15, 17, 20, and 15 after 6-month, 1 year, 3 years, and 5 years, was noted. Surprisingly, a significant decline from a baseline median domain score of 80 to 28, 38, 48, and 48 was also observed in men who underwent nerve-sparing surgery in favorable-risk disease. Similarly, 33% of patients undergoing nerve-sparing RP reported an erection insufficient for penetration at baseline rising to 76% after half a year and then dropping again to 69% after 1 year, 63% after 3 years and 61% after 5 years. In the group of patients undergoing non nerve-sparing RP, 45% of patients reported a baseline erection insufficient for penetration rising to 87% and then dropping again to 83, 80, and 80% over the course of 5 years (15). This is in line with several other studies reporting on the long term outcomes of RP, all demonstrating a clinically meaningful decline in sexual function after surgery (Table 1) (16–22, 27). Despite the fact that different questionnaires, risk groups and even changes in the surgical techniques over time have been applied in these studies, they all are remarkably consistent demonstrating a peak in ED shortly after surgery with some recovery over time but also problems remaining for many men. Indeed, as assessed by Capogrosso et al., the probability of regaining potency after surgery for prostate cancer did not improve over the last decade (28). However, late recovery might be possible. Lee et al. reported a probability of recovering erectile function at 24, 36, and 48 month in patients experiencing erectile dysfunction at 12 month of 22, 32, and 40% (29). Similarly, Mandel et al. reported respective recovery rates of 31% at 24 and 37% at 36 month (30).

**Table 1:** Studies evaluating long-term outcomes after radical prostatectomy.

**Study**	**Crook et al. (16)**	**Resnick et al. (17)**	**Jeldres et al. (18)**	**Zelefsky et al. (19)**	**Donovan et al. (20)**	**Chen et al. (21)**	**Mazariego et al. (22)**	**Hoffmann et al. (15)**
Special features	Low-risk PC only (T1/T2a, GS ≤ 6, PSA < 10 ng/ml)		Low-risk PC only (T1/T2a, GS ≤ 6, PSA < 10 ng/ml)		Most of the operations involved an open retropubic, nerve-sparing approach	86.6% of RP were robotic		Separation into favorable risk cT1 to cT2b, PSA ≤ 20 ng/ml, ISUP 1-2 and unfavorable-risk ≥ cT2c, PSA 20–50 ng/l, ISUP 1–5
Number of men under-going RP	66	1,164	228	220	553	469	NSRP 192/ RP141	NSRP 675/ RP 402
Questionnaire	EPIC-50^a^ (23)	UCLA-PCI^a^ (24)	EPIC-50^a^ (23)	46-item questionnaire^a^ (25)	EPIC-50^a^ (23)	PCSI^b^ (26)	UCLA-PCI^a^ (24) EPIC-26^a^ (14)	EPIC-26^a^ (14)
Sexual function		#		#			#	
Baseline	–	69	62	74	61.4	41.6	76/65	80/70
3 month	–	–	–	35	–	80.8	–/–	–/–
6 month	–	21	–	–	25.7	75.7	–/–	28/15
1 year	–	29	31	–	30.1	73.7	31/21	38/17
2 years	–	32	38	52	33.3	–	38/26	–/–
3 years	–	–	39	–	33.9	–	39/27	48/20
4 years	–	–	–	50	34.3	–	–/–	–/–
5 years	39.22	33	–	–	34.5	–	40/28	48/15
15 years	–	17	–	–	–	–	30/20	–/–
Urinary (incontinence) function		#		#			#	
Baseline	–	95	95	90	91.2	9.7	96/95	100/100
3 month	–	–	–	75	–	45.6	–/–	–/–
6 month	–	57	–	–	80.1	32.3	–/–	73/60
1 year	–	70	80	85	86.5	33.0	78/76	79/67
2 years	–	73	81	–	88.1	–	82/78	–/–
3 years	–	–	80	–	87.9	–	83/79	79/67
4 years	–	–	–	88	88.6	–	–/–	–/–
5 years	88.15	72	–	–	88.9	–	84/80	79/69
15 years	–	65	–	–	–	–	75/73	–/–

*RP, radical prostatectomy; PC, prostate cancer; GS, Gleason Score; PSA, prostate specific antigen; ISUP, International Society of Urological Pathology; NSRP, nerve sparing RP; PC, prostate cancer; EPIC, Expanded Prostate Cancer Index Composite; UCLA-PCI, University of California Los Angeles Prostate Cancer Index; PCSI, Prostate Cancer Symptom Indices*.

a *Questionnair domains: scores range from 0 to 100, with higher scores indicating less symptoms and dysfunction*.

b *PCSI domains: scores range from 0 to 100, with higher scores indicating more symptoms and dysfunction*.

#*Some data have been extracted from a graph and might not be fully accurate*.

### Urinary Continence Function

A second, particularly important adverse effect of RP is urinary incontinence. In the study by Hoffmann et al. a clinically meaningful decline in urinary incontinence function (MCID, 6–9) was shown in men who underwent nerve-sparing RP, from a median domain score of 100 at baseline to 73 at 6 months, with limited subsequent improvement (79 at 3 and 5 years) (scores range from 0 to 100, with higher scores indicating less symptoms and dysfunction). In unfavorable risk disease, men treated with prostatectomy showed a clinically meaningful decline in continence function (MCID, 6–9), with median domain scores falling from 100 at baseline to 60 at 6 month and 69 at 5 years (15). At 5 years, nerve-sparing RP in men with favorable risk disease was associated with a 10% rate of urinary leakage compared to a 16% rate in men with unfavorable risk disease (15). Most of the other observational studies reviewed in this context report intermediate- to long-term results for RP (Table 1) (16–22, 27). Studies that use the EPIC, which provides a more comprehensive assessment of urinary function, report a decline in urinary continence, but less irritative and obstructive voiding symptoms compared to baseline (15, 16, 18, 20). Similarly to what is observed in postoperative ED, post-RP urinary incontinence is multifactorial. In addition to the surgical techniques that are discussed later in this manuscript several preoperative factors such as age, cancer characteristics, prostate size and preoperative lower urinary tract symptoms affect continence rates (31). Studies have shown that continence rates are lower in elderly patients and men with concomitant disease and a high Charlson morbidity index (32). Other factors that may affect postoperative short and long term continence rates are presence of preoperative ED (33), the membranous urethral length (34), the presence of a median lobe (35), previous transurethral resection of the prostate (36), bony pelvic dimension (37), cigarette smoking at the time of surgery (31), and type 2 diabetes mellitus (38).

### Neglected Side Effects

There are a wide range of sexual side effects that affect patients' quality of life but are often overlooked. They include climacturia, arousal incontinence, orgasmic disturbances, and penile anatomical changes (39). Climacturia is defined as involuntary loss of urine in relation to orgasm (40). In a study by Mitchell et al. 22.4% of patients described climacturia as a major problem 3 month after surgery vs. 12.1% 24 month after surgery (41). This time dependent decrease has been reported by other studies as well, although this is not consistent across studies (40, 42–44).

Urinary incontinence during arousal has been reported in 29–49% of sexually active patients following RP and seemed to be associated with severity of daytime urinary incontinence, improving over time (39). Decreased orgasmic sensation has been found in 3.9–70% in selected groups after RP with nerve sparing technique and lower age being protective (45–48) and painful orgasms have been reported in 9.5–14% of all men following RP (46, 49–51). There are numerous studies on penile shortening after RP, but reported rates are inconsistent and range from 0 to 100% (52–55) with nerve sparing surgery, recovery of erectile function, and younger age being predictors of retaining length.

### Surgical Technique

The advent of robotic surgery led to a further evolution of the RP technique. The magnified three-dimensional view and the seven-degree motion provided by the robotic instruments allow for a more precise identification of anatomic structures and were designed to improve patient outcomes. Indeed, in a randomized phase III trial comparing open vs. RARP, patients undergoing RARP had a shorter hospital admission time and less blood loss. However, no differences in functional or oncological outcomes were observed at 12 weeks compared to open RP (56) and follow-up at 24 month confirmed similar functional outcomes with both techniques (57). Another prospective, non-randomized, multicentre trial of 778 patients undergoing open RP and 1,847 undergoing RARP found no statistically significant difference regarding urinary incontinence 12 month after surgery with incontinent rates of 21.3% after RARP and 20.2% after RP. However, RARP resulted in a statistically significant higher proportion of men (30%) with erectile function 12 month after surgery than RP (25%) (58, 59), but further follow up demonstrated similar functional outcomes at 24 month (59–61).

Comparing RARP and LPR, the most recently published multicentre, randomized, controlled, patient-blinded LAP-01 study provides evidence that RARP results in superior early continence rates. At 3 month the continence rates were 54% for RARP and 46% for LPR. Reported erections sufficient for intercourse were 18% in patients undergoing RARP and 6.7% in LPR patients demonstrating a significant benefit in early potency recovery as well, while oncological and morbidity outcomes were similar (62). Likewise, in a small randomized, single-center RARP yielded better functional results compared to LRP throughout the 5 year follow up (63). However, another small randomized, single-center trial did not observe any significant differences in continence at the 12-month evaluation, though time to capability for intercourse was significantly shorter for RARP (64).

Further research focuses not only on the primary technique of RP (open vs. laparoscopic vs. robotic), but also on the exact surgical approach. A recently published Cochrane Review analyzed the standard RARP approach dissecting the so-called space of Retzius anterior to the bladder compared to the Retzius-sparing or posterior approach where the Retzius is left intact (65). Accordingly, the Retzius-sparing approach may improve early continence up to 6 month and improve early urinary quality of life but ultimately results in similar continence outcomes at 12 month (65). Several other surgical techniques like for example anatomic bladder neck preservation (66), posterior reconstruction (“Rocco” stitch) (67, 68), the periurethral suspension stitch (“Patel” stitch) (69), total anatomical reconstruction (70) and suture ligation with suspension of the dorsal venous complex (71) improve early urinary continence, but outcome data beyond 12 month are mostly lacking. Hence, overall, especially when taking long-term outcome data into account, no surgical approach can be definitely recommended over another.

## Discussion

To date men suffering from localized PCa have multiple equally effective treatment options to choose from and only few patients with early stage PCa progress to metastatic disease and die from the disease itself within 10–15 years (72). Men diagnosed with low-risk PCa may be even managed with active surveillance or choose a curative treatment option like RP or radiation. These options have been shown to be equally effective in terms of cancer control at least in the first 10 years after treatment (73, 74).

Thus, paying attention to short- and long-term functional outcomes of treatment is therefore essential to understand the trade-offs between cancer control and adverse treatment effects and to individualize treatment decisions. Indeed, a recently published study evaluating treatment satisfaction and decision regret post RARP in 106 patients demonstrated high regret in one third of patients, associated with worse disease-specific quality of life, sexual and erectile function measures (75). Outcomes following RP, including perioperative, oncologic and health-related quality of life outcomes, are multifactorial. Pretreatment patient and tumor characteristics as well as baseline function play major roles as well as surgeon experience and techniques (76, 77). Whilst major peri- and post-operative complications are rare, men are more frequently suffering from long-term urinary incontinence and ED (15–22, 27). There is a chance of improvement especially in the first few weeks to month after the surgery or even later (29, 30), but some men will be bothered for the rest of their life, not only by incontinence and erectile dysfunction, but also less acknowledged sexual side effects. However, when talking about the sequelae of RP we need to discuss them in the context of other treatment options. Studies comparing RP to other treatment options commonly report men in the prostatectomy group to be more likely to be bothered by urinary incontinence and erectile dysfunction when comparing intermediate-term data. However, men in all treatment groups experienced declines in sexual function over time, including those who underwent active surveillance. This decline was in part due to progression to treatment and in part due to age-related functional changes. Moreover, Litwin et al. applied the University of California Los Angeles Prostate Cancer Index (UCLA-PCI) to a population of 598 men without prostate cancer and found, that 50% were unable to achieve an erection sufficient for intercourse and 32% were unable to achieve an erection sufficient for any sexual activity. Urinary incontinence was reported in 31% of men, with at least weekly urinary incontinence reported in 18% (78).

## Conclusion

RP is a common treatment for PC and can cure many patients. However, despite many advancements in technique, long-term post-surgical decline in erectile function and urinary continence, and other less frequent side effects can affect a relevant proportion of men. This effect on health-related quality of life needs to be taken into account when counseling patients about their treatment options.

## Author Contributions

CK and GG contributed to conception and design of the manuscript. All authors contributed to the article and approved the submitted version.

## Conflict of Interest

The authors declare that the research was conducted in the absence of any commercial or financial relationships that could be construed as a potential conflict of interest.
